# Morphological Characteristics of the Distal Iliopsoas Tendon and Its Relationship to Adjacent Osseous Structures

**DOI:** 10.7759/cureus.101395

**Published:** 2026-01-12

**Authors:** John Tracey, Jon Cornwall, Leigha M Lynch, Heather F Smith

**Affiliations:** 1 Department of Anatomy, Midwestern University Arizona College of Osteopathic Medicine, Glendale, USA; 2 Anatomy, University of Otago, Dunedin, NZL; 3 Anatomy, Midwestern University, Glendale, USA

**Keywords:** anatomical variation, anterior superior iliac spine, lesser trochanter, psoas major, psoas minor

## Abstract

Background

The iliopsoas muscle influences lumbo-pelvic posture and gait stability, with its distal portions contributing to common anterior hip pathologies, including iliopsoas bursitis, impingement, and tendinopathy. Despite its role in such pathologies, the anatomical variation of the iliopsoas musculotendinous morphology is poorly described. This project, therefore, evaluated distal iliopsoas tendon morphology and its relationship to pelvic osseous structures.

Methods

Data on the iliopsoas tendon and osseous morphology were collected from 49 body donors. Linear measurements were collected between the anterior superior iliac spine (ASIS), lesser trochanter (LT), pubic tubercle (PT), and medial and lateral femoral epicondyles (ME, LE). Angles between landmarks were calculated trigonometrically. Correlation analyses were performed to determine whether correlations existed among variables, and independent t-tests and Chi-squared tests were used to assess differences among sexes and tendon morphotype categories.

Results

Tendons were classified into three morphotypes: notched (53%), fan-shaped (25%), and narrow (22%), with narrow tendons being more common in males (25.8% versus 16.7%) and fan-shaped tendons in females (33.3% versus 19.4%). Fan-shaped tendons had a higher mean LT angle, but no variables differed significantly between tendon types. Females had significantly higher LT angle (*p* = 0.007) and psoas minor presence (60% versus 32.4%) versus males. Highly significant correlations were identified between ASIS angle and length from PT to LT (*r* = 0.659; *p *< 0.001), LT angle and length from ASIS to PT (*r* = 0.581; *p* < 0.001), and PT angle and distance between ASIS and LT (*r *= 0.474, *p* = 0.001). Overall, there was a significant relationship between sex and several linear and angular measurements.

Conclusion

The identification of distinct tendon morphotypes and relationships between the musculotendinous complex and osseous landmarks provides novel insight into anterior hip morphology. Findings may assist in the identification of persons at risk of anterior hip pathology.

## Introduction

The etiology of musculoskeletal pain affecting the anterior hip and pelvic region is varied, with common anterior hip pathologies of musculoskeletal origin being responsible for a multitude of chronic sequelae that have a lasting impact beyond the initial disease presentation [1-2]. Hip pain is estimated to affect approximately 10% of the general population and may be aggravated by basic functional movements, leading to chronic pain and impaired functional mobility [3]. Delay in diagnosis due to vague symptomology and poor localizing signs can further compound these problems. Risk factors for hip pain include such demographics as female gender, increased age (>75), white racial status, obesity, lower socioeconomic status, and previous hip injury [4]. Furthermore, physiological changes have been documented to occur during pregnancy with respect to the anterior hip and pelvis, such that pregnant patients show decreased hip flexion, extension, and abduction with increased hip external rotation [5]. These changes have a significant impact on measured gait analysis, underscoring the importance of anatomical variation of the psoas major and its attachments in the development of anterior hip pain in peri- and post-partum patients [5].

Hip pain is a common chronic pathology affecting a significant proportion of adult patients [3]. The overall financial impact of chronic pain in the United States, considering healthcare-related costs and secondary costs due to lost productivity, was greater than the financial impact of cardiovascular disease, cancer, and diabetes in 2010 and is projected to continue to rise in the future [6]. A better understanding of the factors involved in the pathogenesis of these pathologies is important to facilitate the development of strategies to allow accurate diagnosis, facilitate targeted rehabilitation, and direct appropriate interventions. This is particularly the case for pathologies involving the psoas musculotendinous unit, where limited data on morphology currently exist, and a specific understanding of the morphological variation of this structure is limited. Common pathologies involving the psoas musculotendinous unit that affect the anterior hip region include iliopsoas bursitis, impingement, tendinopathy, and psoas syndrome.

Iliopsoas bursitis is defined as inflammation and enlargement of the iliopsoas bursa [7], while iliopsoas impingement refers to inflammation and pain in the iliopsoas muscle resulting in abnormal movement of the hip. Both conditions are common causes of secondary hip disorders, including anterior hip pain, labral tears, acetabular articular cartilage damage, and idiopathic arthritis [8]. There are difficulties around the diagnosis of both conditions, which can increase and prolong impacts on patients due to delays in treatment and symptom relief [9]. Previous studies have shown that a combination of imaging and fluid bursa aspiration is key to a timely diagnosis for bursitis [10], while research has implicated scarring of the iliopsoas tendon in relation to impingement as a direct mechanism for the development of labral tears and associated anterior hip pain in patients [11]. Psoas syndrome results from overuse or trauma to the iliopsoas muscle and commonly presents as groin pain in young athletes or dancers [12]. The syndrome of pain symptoms is described interchangeably with iliopsoas tendinitis and impingement, and is often seen secondarily to concomitant hip pathologies such as total hip arthroplasty (THA) and iliopsoas bursitis [12].

The degree to which iliopsoas morphology may contribute to this collection of pathologies has not previously been elucidated, and despite the prevalence of anterior hip pathologies involving the psoas musculotendinous unit, few studies have explored the morphology of this region in any detail. Such data are important as it is unclear whether variations in tendon morphology exist. This information is relevant as it contributes to our ability to determine whether certain people may be predisposed to anterior hip pathology. This project, therefore, aimed to investigate the morphology of the iliopsoas tendon, focusing on variation in the location of the lesser trochanter in relation to adjacent osseous structures, as well as the precise location of the insertion and the morphology of the iliopsoas tendon. This study aimed to shed light on how variation within adult structures of the proximal pelvic/femoral musculoskeletal unit, which may be related to potential anterior hip pathology.

This article was previously presented as a meeting abstract at the 2025 American Association for Anatomy Annual Scientific Meeting on March 29, 2025.

## Materials and methods

Access to body donors

Forty-nine human body donors (formalin-fixed cadavers) from Midwestern University’s Body Donation Program were utilized in this study and were evaluated for tendon morphotype. A subset of 44 of these donors included sufficient anatomical detail to be included in the quantitative portion of the study. Inclusion in the study was determined primarily by the quality of the dissections, which were performed by first-year medical students as part of their anatomy coursework. All donors were included whose dissections permitted adequate evaluation of the anatomical structures of interest. Dissection and data generation were performed by one investigator (John Tracey), and data were collected bilaterally whenever possible. Donor ages at death ranged from 40 to 98, with a mean age of 80.5 years. Additional demographic information, including height, weight, and BMI, was not readily available from donor records. This study was determined to be IRB-exempt by the Midwestern University Institutional Review Board because the project does not meet the definition of human subjects research as defined in 45 CFR 46.102.

Data collection

**Figure 1:**
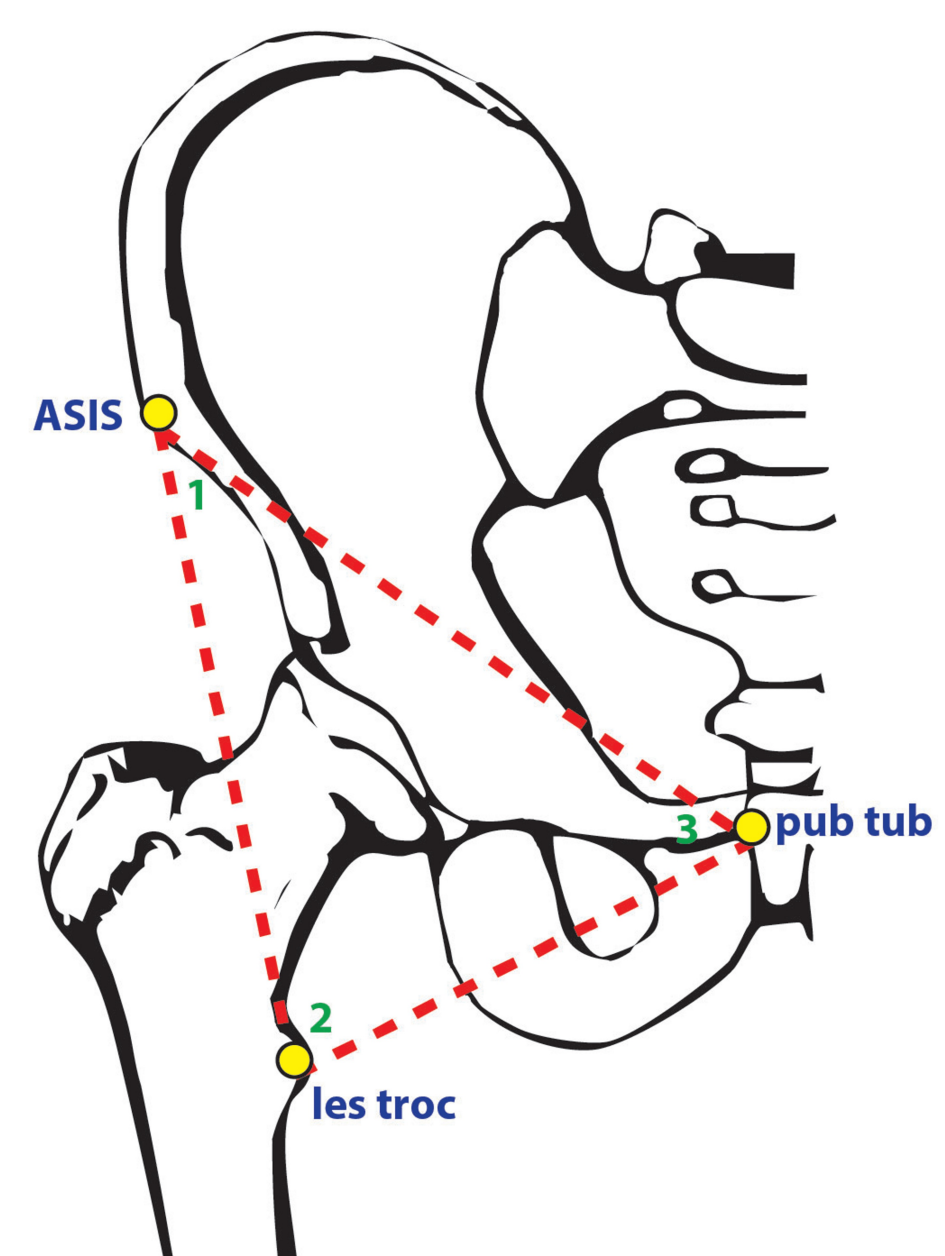
Schematic illustration identifying anatomical landmarks (yellow circles) and angles (between red dotted lines; angles labelled 1-3) utilized in the present study from the pelvis. Abbreviations: ASIS: anterior superior iliac spine; les troc: lesser trochanter; pub tub: pubic tubercle. 1 = angle from ASIS; 2 = angle from lesser trochanter; 3 = angle from pubic tubercle Image adapted from Netter [13]

Blunt dissection of hip adductor muscles, including pectineus, adductor longus, adductor brevis, and sartorius, was performed to facilitate identification of the lesser trochanter (LT). Once the LT was identified, proximal dissection of overlying muscles and vascular structures, including the femoral artery and vein, was performed with care not to disrupt the iliopsoas tendon. Observations on the gross appearance and morphology of the iliopsoas tendon were then recorded. The anterior superior iliac spine (ASIS), pubic tubercle (PT), and medial femoral epicondyle (ME) were identified to facilitate the acquisition of linear measurements. These were taken between ASIS and LT, ASIS and PT, ASIS and lateral epicondyle (LE), ASIS and ME, and PT and LT (Figure 1).

These osseous pelvic landmarks were chosen to facilitate a proxy measure of pelvic symmetry that could be used to assist in the interpretation of any differences in tendon morphology, which have not been previously described. Measurement to the ME was performed to allow a proxy measure for the influence of donor height. The presence or absence of the psoas minor muscle was determined through dissection and visual examination of the posterior abdominal wall and anterior surface of psoas major and documented.

Data analyses

Statistical analyses of quantitative measurements were conducted unilaterally (left side when possible) to avoid redundancy. Trigonometric calculations were performed to determine the angles within the scalene triangle formed between ASIS, LT, and PT using an arc cosine function. Correlation analyses were performed to determine whether significant statistical associations existed between the variables of interest, such as the linear distances measured between the ASIS, LT, PT, ME, and LE, and the calculated angles from the ASIS, LT, and PT to determine if a relationship existed between these variables outside of the trigonometric relationships expected from these calculations. Partial correlation analyses were also conducted, treating sex as a covariate to evaluate whether the correlations differed between the sexes. Independent sample t-tests were employed to assess whether significant differences existed in the linear and angular variables between the sexes, between individuals with and without psoas minor, and among tendon morphotype categories. Chi-squared analyses were used to test whether iliopsoas tendon classification frequencies and the presence of psoas minor differed between the sexes. Sequential Bonferroni corrections were applied to control for multiple comparisons based on a critical alpha level of p < 0.05. Trigonometric calculations were conducted in Excel (Microsoft, Palo Alto, CA), and other analyses were conducted using IBM SPSS Statistics for Windows, Version 27.0 (released 2019, IBM Corp., Armonk, NY).

## Results

Tendon morphotypes

**Figure 2:**
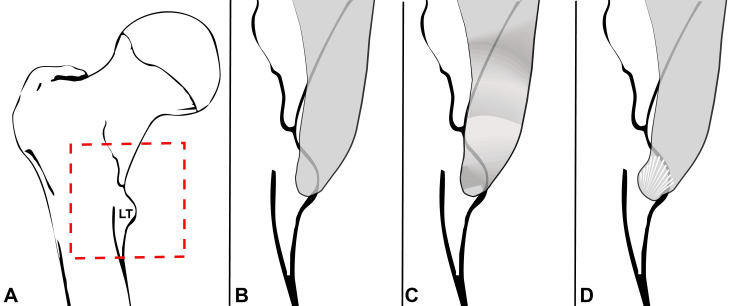
Illustration of iliopsoas tendon morphotypes identified in this study. A) Overview of proximal femur (anterior view) showing position of magnified areas in B-D (red square). B) Narrow iliopsoas tendon morphotype. C) Notched iliopsoas tendon morphotype. D) Fan-shaped iliopsoas tendon morphotype. LT: lesser trochanter Images created by Dr. Smith H (author) using MS Excel (Microsoft Corp., USA) and Adobe Photoshop (Adobe Inc., USA)

**Figure 3:**
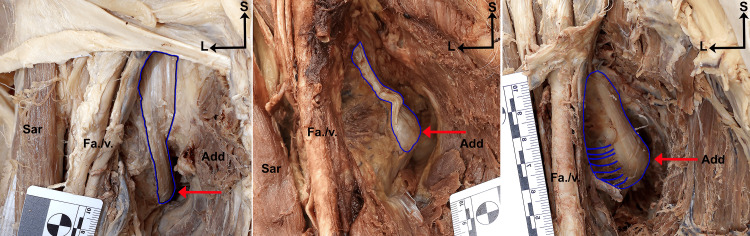
Anterior view of right hip regions showing photographic examples of the iliopsoas tendon morphotypes identified in this study. A) Narrow iliopsoas tendon morphotype. B) Notched iliopsoas tendon morphotype. C) Fan-shaped iliopsoas tendon morphotype. Red arrow points to the lesser trochanter in all panels. Blue outline surrounds the tendon at its insertion onto the lesser trochanter. Abbreviations: Add: adductor muscles; Fa./v.: femoral artery and vein; L: lateral; Sar: sartorius muscle; S: superior Narrow tendons have a single or dual course of bands of tissue running vertically, deep to the anterior femoral muscle mass that insert onto or proximal to the lesser trochanter. Notched tendons show an observable horizontal divot within the course of the tendon proximal to the tendinous insertion at or around the lesser trochanter. Fan-shaped tendons have a large tendinous insertion that spans across the entirety of the surface of the lesser trochanter.

Categorization of the iliopsoas tendon morphotype fell into one of three main categories: narrow, notched, or fan-shaped (Figure 2). Narrow tendons were observed to have a single or dual course of bands of tissue running vertically, deep to the anterior femoral muscle mass that inserted onto or proximal to the lesser trochanter (Figures 2A, 3A). Notched tendons showed an observable horizontal divot within the course of the tendon proximal to the tendinous insertion at or around the lesser trochanter (Figures 2B, 3B). Fan-shaped tendons were observed to have a large tendinous insertion that spanned across the entirety of the surface of the lesser trochanter (Figures 2C, 3C).

Approximately half the sample tendons (including bilateral data) were notched 26 (53.1%), while the narrow and fan-shaped categories each accounted for approximately one-quarter of the sample, 11 (22.4%) and 12 (24.5%), respectively (Table 1). The relative frequencies of each tendon type were similar between sexes (Table 1), although the fan-shaped tendon was found in a slightly higher frequency in female donors (6/18 = 33.3% vs. 6/31 = 19.4%; not significant).

**Table 1:** Frequencies of the three identified iliopsoas tendon morphotypes in the study sample separated by sex.

	Narrow	Notched	Fan-shaped	Total
Females	3 (16.7%)	9 (50%)	6 (33.3%)	18
Males	8 (25.8%)	17 (54.8%)	6 (19.4%)	31
Total	11 (22.4%)	26 (53.1%)	12 (24.5%)	49

Quantitative analyses

Descriptive statistics for the study sample are provided in Table 2. On average, males had higher values for the linear measurements than females. However, in the angle measurements, females had a higher mean angle from the lesser trochanter and lower values in the other two angle measures (Table 2).

**Table 2:** Descriptive statistics for the linear measurements and angles associated with the psoas major and lesser trochanter in the study sample. Linear dimensions provided in cm. *Asterisks and bolded values indicate t-test results between sexes that are significant after a Bonferroni correction. Abbreviations: ASIS: anterior superior iliac spine; E: lateral epicondyle; LT: lesser trochanter; ME: medial epicondyle; PT: pubic tubercle; SD: standard deviation

Variable	Mean: Entire sample	Mean: Females	Mean: Males	SD	Range	Variance	t-test (p-value)
ASIS- ME*	48.9	46.5	51.1	3.9	37.8-57.7	15.2	-4.728 (p<0.001)
ASIS- LE*	49.1	46.6	51.4	3.9	37.6-57.8	15.2	-5.224 (p<0.001)
ASIS- PT	13.0	12.9	13.0	1.3	9.3-15.6	1.7	-0.397 (p=0.347)
ASIS- LT*	16.6	15.9	17.2	1.5	12.8-19.6	2.2	-2.952 (p=0.003)
PT- LT*	10.6	10.1	11.0	1.2	7.7-13.2	1.4	-2.581 (p=0.007)
Angle ASIS	39.7°	39.5°	39.8°	4.7°	28.9-49.9°	22.1	-0.174 (p=0.431)
Angle LT*	51.5°	54.2°	49.1°	6.9°	40.0-67.1°	47.6	2.578 (p=0.007)
Angle PT	88.9°	86.3°	91.1°	8.8°	70.5- 113.3°	77.4	-1.862 (p=0.035)

The correlation analyses revealed significant pairwise relationships between all the linear variables, whether or not sex was controlled for. However, the calculated angles were significantly correlated with only a subset of linear dimensions (Table 3). Specifically, the angle from the ASIS was significantly correlated with the distance between the PT and LT (r = 0.623, p < 0.001), angle from LT was significantly correlated with distance from ASIS to PT (r = 0.518, p < 0.001) and ASIS to LT (r = 0.448, p = 0.002), and angle from PT was significantly correlated with ASIS to LT (r = 0.528, p < 0.001), ASIS to LE (r = 0.410, p = 0.005) and ASIS to ME (r = 0.391, p = 0.008). However, in the partial correlation analyses controlling for sex, the angle from the ASIS was significantly correlated with only the distance from the PT to LT (r = 0.659, p < 0.001), the angle from the LT was only significantly correlated with the distance from the ASIS to the PT (r = 0.518, p < 0.001; Table 3), and the angle from the PT was only significantly correlated with the distance from the LT to the ASIS (r = 0.474, p = 0.001) (Table 3). Donor age at death was not significantly correlated with any of the linear or angular variables.

**Table 3:** Correlation analysis values of statistical significance between angles overlying osteological structures of interest and the collected linear measurements. Abbreviations: ASIS: anterior superior iliac spine; LE: lateral epicondyle; LT: lesser trochanter; ME: medial epicondyle; PT: pubic tubercle Bolded values significant after sequential Bonferroni correction.

	Standard correlation		Partial correlation: Controlling for sex	
Variable	r-value	p-value	r-value	p-value
Angle from the ASIS:				
ASIS - ME	-0.297	0.048	-0.385	0.010
ASIS - LE	-0.260	0.085	-0.353	0.019
ASIS - PT	-0.147	0.336	-0.149	0.335
ASIS - LT	-0.328	0.028	-0.372	0.013
PT - LT	0.623	<0.001	0.659	<0.001
Angle from the LT:				
ASIS - ME	-0.296	0.048	-0.108	0.483
ASIS - LE	-0.344	0.021	-0.160	0.300
ASIS - PT	0.518	<0.001	0.581	<0.001
ASIS - LT	-0.448	0.002	-0.351	0.019
PT - LT	-0.252	0.095	-0.136	0.378
Angle from the PT:				
ASIS - ME	0.391	0.008	0.297	0.050
ASIS - LE	0.410	0.005	0.318	0.035
ASIS - PT	-0.328	0.028	-0.359	0.017
ASIS - LT	0.528	<0.001	0.474	0.001
PT - LT	-0.136	0.373	-0.264	0.084

**Figure 4:**
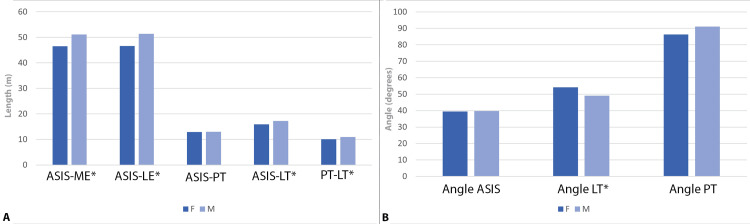
Bar plots showing mean values for variables in both sexes in the study sample. A) Linear dimensions (in cm); B) angle measures (in degrees). Abbreviations: ASIS: anterior superior iliac spine; LE: lateral epicondyle; F: female; LT: lesser trochanter; M: male; ME: medial epicondyle; PT: pubic tubercle Image created by Dr. Smith H (author) using Adobe Photoshop (Adobe Inc., USA)

In addition, the statistical analyses revealed significant sex differences in the following linear dimensions: ASIS to ME (t = -4.728, p < 0.001), ASIS to LE (t = -5.224, p < 0.001), ASIS to LT (t = -2.952, p = 0.003), and PT to LT (t = -2.581, p = 0.007) (Table 3, Figure 4). We then evaluated whether significant differences in linear measurements would translate to significant differences in the angles calculated from such measurements and found statistically significant variation in the angle from the LT (t = 2.578, p = 0.007). There were no significant differences in any of the linear or angle measurements among the tendon categories. Raw data are provided in the Appendix. 

Presence of the psoas minor

Finally, in addition to the measured and calculated quantitative data, categorical data on the presence or absence of the psoas minor were analyzed. Of the 77 specimen sides that could be evaluated for the psoas minor, 30 possessed a psoas minor (39.0%), while 47 (61.0%) did not. Of these, the Chi-squared analysis revealed that psoas minor was found significantly more frequently in females (18/30; 60.0%) than in males (12/37; 32.4%) (p = 0.024). Dividing the subjects into two groups, those with a psoas minor and those without, we found no statistical association between the presence of the psoas minor and any of the measured linear or angular variables. The mean angle from the LT in donors in which the psoas minor was present was 10.26° larger than the mean angle from the LT in donors without a psoas minor present, although this difference was not statistically significant. There was no statistical association between tendon type and the presence of psoas minor. 

## Discussion

This study aimed to investigate the morphology of the iliopsoas tendon and osseous structures, and in doing so, it documented variation in the location of the lesser trochanter, the morphology of the iliopsoas tendon, and the location of the insertion of the iliopsoas tendon onto and adjacent to the LT. Through the collection and analysis of both quantitative data in the form of linear measurements of proximal femoral and pelvic osteological features and qualitative classification of the morphotype of the iliopsoas tendon when possible, we sought to define the precise position of the LT and document the form and insertion of the iliopsoas tendon, respectively. Tendon morphotypes were classified as narrow, notched, or fan-shaped, with narrow representing the historically described morphology of the iliopsoas tendon, while notched and fan-shaped tendons showed distinct variations from the classically described anatomy of the region (e.g., [13]). These variations may reflect pathological changes or simply anatomical variation to the tendon and warrant further investigation as potential indicators of chronic disease processes to the anterior hip and iliopsoas tendon. Variations in the calculated pubofemoral angles (ASIS, LT, and PT) and their relation to overall stature may highlight novel structural and gender related trends in anterior hip musculoskeletal structure. The presence of a key musculotendinous structure in the anterior hip, psoas minor, may have tangential effects on the underlying pubofemoral skeleton.

Morphological variation of the iliopsoas tendon

We observed three major morphological categories of iliopsoas tendon insertions: narrow, notched, and fan-shaped. Morphologic variation of the iliopsoas tendon and its relationship to the presence of iliopsoas tendon pathology, specifically bursitis, has not been widely studied outside of its description as a distension of the iliopsoas muscle bursa due to synovial fluid and/or hypertrophic synovium [10]. However, variation in the number of tendinous bands of tissue (one, two, or three) and the location of insertion of the iliopsoas tendon has been previously reported, and variation has been observed in over 50% of a recent study’s specimens [14], further reinforcing the existence of variation in these structures.

Recent retrospective research on surgical outcomes following THA has shown a potential reduction in anterior hip pain and post-operative iliopsoas tendinitis through the use of anatomically contoured femoral heads, in contrast to large diameter femoral heads, which may impinge upon surrounding structures [15]. Given the variation in tendon morphotypes observed in this study, it is possible that the morphology and insertion of the iliopsoas tendon itself are an independent risk factor for the occurrence of these iatrogenic findings following THA. Further work is required to evaluate this relationship between tendon morphotype and THA surgical outcomes to determine whether a causal association exists. Furthermore, the variation we observed in notched tendon morphotypes may be correlated with an increased risk of impingement due to the greater freedom of motion within the iliopsoas tendon. Iliopsoas tendinopathy is particularly related to THA [16], and as such, potential risk factors for the development of hip pathology, such as morphological variation of the musculotendinous unit, should be considered prior to interventional procedures. 

Outcomes around pubofemoral angles

Findings revealed a strong correlation between the linear measurements collected and the calculated pubofemoral angles. However, much of that significance can be attributed to the trigonometric principles utilized to calculate the pubofemoral angles. The only statistically significant relationship we noted between variables outside the immediate triangle created to calculate the pubofemoral angles (Figure 1) was between the angle from the PT and the linear distance measured from the ASIS to the ME and LE, respectively. The impact of these findings is difficult to interpret and warrants further investigation. To what degree pelvic morphology, in terms of width, height, and depth, and overall human stature, are interrelated has not been thoroughly described. Simon and colleagues [17] previously demonstrated that the linear measurements of long bones are highly correlated with stature (r ≥ 0.76), building on the previous work of [18], who created linear regression formulae to infer stature based on long bone measurements. Our findings may indicate the potential for a relationship between pelvic measurements and femur length, as suggested by the significant correlations among several tested variables, but they were not sufficiently significant for us to draw informed conclusions.

Sex specific differences in the calculated pubofemoral angles were also noted, with males having, on average, larger pubofemoral angles, as well as measured linear distances between osteological points of interest (ASIS, LT, PT). However, a statistically significantly larger pubofemoral angle was noted from the LT for our female donors specifically, and was the only measurement (linear distance or pubofemoral angle) in which female donors had a larger average measurement than male donors. The importance of this finding may be two-pronged, in both young athletic female patients and pregnant or post-partum women.

Iliopsoas bursitis is traditionally associated with active, younger individuals and is more common in female patients with symptoms exacerbated by increased physical activity [19], suggesting that the femoral angle is perhaps important in the etiology of symptoms. Data from this study suggest that the location of the LT and the attachment of the psoas major muscle are related to angles within the pelvic femoral unit, which in turn would potentially influence whether there is a mechanical compromise (e.g., against osseous landmarks) of structures such as the iliopsoas tendon.

Furthermore, physiological and morphological changes that occur during pregnancy have been well documented, with lower back pain being cited as a common complaint, secondary to hormonally driven structural changes in the lumbar spine (increasing lumbar lordosis) and pelvis (increasing SI joint and pubic symphysis mobility) [20]. Anterior hip pain in pregnancy is also common, with some estimates indicating that over 50% of pregnant women experience hip or lower back pain [21]. However, these studies focused on labral pathology and not the iliopsoas tendon as the primary mechanism for anterior hip pain in this population. A greater angle measured from LT may allow for greater iliopsoas joint mobility, especially during hormonally driven peripartum structural changes. The course of the psoas major and its contribution to the iliopsoas tendon further indicate the potential for the development of anterior hip pathology involving the iliopsoas tendon secondary to changes occurring in the lumbar spine. While our study did not specifically address these points, our findings call into question the presumed etiology of anterior hip pain in the peri- and postpartum periods and indicate the need for further research into this phenomenon.

Incidence of the psoas minor and relationship to other variables

The psoas minor is often described as a vestigial muscle in humans, with reported incidence ranging from 40% to 56% in different study populations [22]. It has previously been described as having a fusiform shape extending from its origin on the T12-L1 lateral vertebral bodies to its insertion on the pecten pubis and iliopectineal arch. Our study revealed that donors who possessed a psoas minor had, on average, a greater angle from the LT by 10.3°; however, this difference was not statistically significant. Neumann and Garceau [23] previously catalogued morphological variation in psoas minor and hypothesized that variation in the distal attachment of the muscle onto the iliac fascia may influence the stability, and thereby insertion, of the underlying iliopsoas musculature, and that this may be clinically related to inflammation and pathology involving the iliopsoas muscle and adjacent anterior hip tissue. Patra and colleagues [22] also called into question the influence that morphological variation within the psoas minor at its attachment onto the iliac fascia may have on the underlying iliopsoas position, mechanical stability, and the development of inflammatory changes to the musculotendinous unit at large. The true influence of the psoas minor on anterior hip pathology has yet to be illuminated, and future research is necessary to parse out the degree to which this muscle contributes to said pathology.

Potential relevance to lumbar spine pain

Previous research has documented the contribution of the iliopsoas muscle in the onset of chronic back pain [24] and evaluated morphological differences in psoas major morphology in patients with multilevel spinal stenosis [25]. However, an investigation into the role of anatomical variation of the iliopsoas tendon and osseous morphology in the pathogenesis of back pain is currently nonexistent. While not the focus of this research project, the role of chronic back pain and its relationship to musculoskeletal pain within the hip and lower extremities should not be overlooked when evaluating patients who initially report chronic pain without a clear etiology for anterior hip symptoms. Chronic back pain remains one of the most common complaints that patients report to their medical providers, and it has a wide range of potential causes [26]. Hip and lower back pain are common concomitant pathologies, and the diagnosis of reported hip pain can often be made more difficult by the association of concomitant lumbar spine and knee joint pain, often reported by these patients [3]. Studies have shown that THA can have a significant reduction in lower back pain post-operatively, although the mechanism is not well understood [27,28], while THA can also provide significant improvement in lumbar flexibility associated with decreased pain [29]. The finding of different tendon morphologies and novel data on osseous landmarks may assist with further explorations of potential interactions between lumbar spine and hip pathologies.

Study limitations

This study was potentially limited by several factors that influenced the course of the project. First, data collection was confined to those donors within the Midwestern University Body Donation Program and was therefore not a true random sampling of the general population. However, donors to this program typically exhibit characteristics of wide-ranging backgrounds, which reduces the confounding effect of investigating only a single population of patients. Second, data collection occurred following first year anatomical dissection by medical, nurse anesthetist, and physician assistant students. While the structures of interest for this paper were reliably located and identified as undamaged, any potential impact this prior dissection may have had on the structures cannot be eliminated completely. Third, donors underwent a pelvic hemi-section prior to the outset of data collection that removed the right lower extremity by cutting vertically through the pelvis and sacrum. However, the osteological points of interest, namely ASIS and the pubic tubercle, were preserved in every donor included in the sample (a single donor was excluded based on the laterality of the hemi-section removing the pubic tubercle from the right lower extremity unit) and were reliably identified during data collection.

Applications to orthopedic pathology

Orthopedic pathology is a broad category of clinical disease with many contributing factors leading to the development of individual diagnoses. Understanding the associated morphological variation is important for accurate diagnosis, rehabilitation, and intervention. Early identification of at-risk groups for anterior hip pathology would assist the provision of timely intervention and prophylaxis so patients may avoid the more deleterious effects of long-term disease processes. The benefit of early intervention and mobilization during treatment of musculoskeletal injury has been documented with earlier return to activity decreasing overall recovery time and decreasing the need for more costly and invasive interventions in the future [30,31]. More recently, chronic orthopedic pathology such as osteoarthritis is being viewed as a continuum of disease with the focus shifting towards early treatment and prevention rather than conservative management until the inevitable arrival at definitive surgical treatment [32,33]. These factors highlight how clinical management of musculoskeletal disease can benefit from a thorough understanding of anatomical variation and its application to the identification of those patients at the greatest risk for the development of these pathologies.

## Conclusions

This project examined distal iliopsoas tendon morphology and its relationship to pelvic osseous structures. Research focused on iliopsoas morphological variation can help identify variation that exists within patients and inform clinicians as they work to evaluate and diagnose the pathology underlying anterior hip pain. Whether the morphological variations observed may relate to the presence or absence of common iliopsoas tendon pathologies (e.g., bursitis, impingement, psoas syndrome) is unclear. However, future research should continue to explore the relationship between the psoas musculotendinous unit and the pelvis to provide insight as to whether or how these structures play a role in the pathogenesis of the most common anterior hip pathologies.

## Appendices

**Table 4:** Complete table of raw data from specimens collected in this study.

Donor # (side)	Sex	Age	Tendon morphotype	Tendon type #	ASIS - Med epicondyle (cm)	ASIS - Lat epidcondyle (cm)	ASIS - Pub Tub (cm)	ASIS - lesser troch (cm)	Pub Tub - lesser troch (cm)	Angle of triangle from ASIS - numerator	Angle of triangle from ASIS - denominator	Angle of triangle from ASIS (degrees)	Angle of triangle from LT - num	Angle of yriangle from LT - Den	Angle of triangle from LT (degrees)	Angle of triangle from PT - Num	Angle of triangle from PT - Den	Area of triangle from PT (Degrees)	Psoas minor (?)
1 (L)	F	98	Unknown	?	48	49	14	17	12	341	476	44.24305212	237	408	54.4873734	51	336	81.26957448	N
1 (R)	F	98	Unknown	?	48	48.5	13.5	16.1	11.7	304.57	434.7	45.5212951	213.85	376.74	55.41467839	59.93	315.9	79.06402651	N
2 (L)	F	87	Unknown	?	42.7	42.7	13.1	14.3	11.6	241.54	374.66	49.85747434	167.44	331.76	59.68841464	101.68	303.92	70.45411101	N
2 (R)	F	87	Unknown	?	44	43.5	13.8	13.8	12.4	227.12	380.88	53.3944029	153.76	342.24	63.30279855	153.76	342.24	63.30279855	N
3 (L)	M	98	Notched	2	49.6	49.1	15.6	16.9	13.2	354.73	527.28	47.71998287	216.49	446.16	60.97248936	131.99	411.84	71.30752777	N
3 (R)	M	98	Unknown	?	49.7	49.3	15.5	16.9	15.3	291.77	523.9	56.15698939	279.45	517.14	57.29076741	188.73	474.3	66.55224319	N
4 (L)	F	90	Narrow	1	47.6	46.3	13.3	15.3	9.3	324.49	406.98	37.12583031	143.69	284.58	59.67398923	29.29	247.38	83.20018046	Y
4 (R)	F	90	Unknown	?	46.9	45.6	13.2	14.5	9.3	298	382.8	38.87889572	122.5	269.7	62.98598845	50.48	245.52	78.13511583	Y
5 (L)	M	63	Unknown	?	51.7	50.6	11.9	15.8	10.8	274.61	376.04	43.09112911	224.67	341.28	48.82845135	8.61	257.04	88.08041954	N
5 (R)	M	63	Unknown	?	51.7	52.6	14.2	18.4	10.6	427.84	522.56	35.04128678	249.28	390.08	50.27910235	-24.56	301.04	94.67961087	N
6 (L)	F	80	Unknown	?	43.7	43.7	11.5	12.8	9.2	211.45	294.4	44.09059762	116.23	235.52	60.42886667	53.05	211.6	75.48053572	Y
6 (R)	F	80	Unknown	?	45.4	44.7	11.3	14.5	9.6	245.78	327.7	41.40830041	174.72	278.4	51.12773907	9.6	216.96	87.46396052	Y
7 (L)	M	87	Unknown	?	51.8	51.1	13.7	17.4	9.9	392.44	476.76	34.5996815	213.08	344.52	51.79451035	-17.06	271.26	93.60580816	N
7 (R)	M	87	Unknown	?	49.9	50.2	12.3	17.3	10.8	333.94	425.58	38.3098017	264.64	373.68	44.91138776	-31.36	265.68	96.77881054	N
8 (L)	F	49	Unknown	?	51.3	50.6	12.4	17	10.4	334.6	421.6	37.47270433	243.4	353.6	46.50048652	-27.08	257.92	96.02680915	N
8 (R)	F	49	Unknown	?	52.6	51.9	13.3	17.4	9.6	387.49	462.84	33.15430331	218.03	334.08	49.25995534	-33.71	255.36	97.58574134	N
9 (L)	F	94	Unknown	?	47.6	46.6	11.9	14.2	9.3	256.76	337.96	40.55916894	146.52	264.12	56.30665149	26.46	221.34	83.13417957	Y
9 (R)	F	94	Unknown	?	46.7	46	11.6	13.8	9.2	240.36	320.16	41.34464548	140.52	253.92	56.39923663	28.76	213.44	82.25611789	Y
10 (L)	M	83	Unknown	?	52.5	51.8	12.4	18.6	9.8	403.68	461.28	28.93962656	288.24	364.56	37.75354019	-96.16	243.04	113.3068333	Y
10 (R)	M	83	Unknown	?	51.1	49.8	13.1	18	9.7	401.52	471.6	31.63578643	246.48	349.2	45.10239796	-58.3	254.14	103.2618156	N
11 (L)	M	90	Unknown	?	52.2	52.4	10.6	15.2	9.5	253.15	322.24	38.22429614	208.93	288.8	43.66061981	-28.43	201.4	98.11508405	Y
11 (R)	M	90	Notched	2	52.2	52.4	12.6	16.4	10.8	311.08	413.28	41.17432271	226.84	354.24	50.18156817	6.44	272.16	88.64410912	N
12 (L)	F	82	Unknown	?	46.2	45.6	11.9	15.1	9.5	279.37	359.38	38.97991006	176.65	286.9	51.99576182	3.85	226.1	89.02432812	Y
12 (R)	F	82	Other	?	45.2	45.6	12.3	15.9	9.9	306.09	391.14	38.50455408	199.53	314.82	50.66964667	-3.51	243.54	90.82579925	Y
13 (L)	M	97	Notched	2	53.2	52.7	13.3	17.9	10.1	395.29	476.14	33.88101992	245.53	361.58	47.23074283	-41.51	268.66	98.88823724	N
13 (R)	M	97	Unknown	?	52.4	51.7	14.2	19.7	11.3	462.04	559.48	34.32642786	314.14	445.22	45.12327585	-58.76	320.92	100.5502963	N
14 (L)	M	80	Unknown	?	53.6	53.3	11.8	17.1	10.8	315.01	403.56	38.68648606	269.81	369.36	43.07337183	-36.53	254.88	98.24014211	N
14 (R)	M	80	Notched	2	53.1	52.6	12.6	17.6	10.2	364.48	443.52	34.73568507	255.04	359.04	44.73752198	-46.96	257.04	100.526793	N
15 (L)	F	60	Narrow	1	37.8	37.6	10.8	13.1	8.9	209.04	282.96	42.37396522	134.18	233.18	54.86987876	24.24	192.24	82.75615602	N
15 (R)	F	60	Notched	2	38.5	38.9	10.8	14.2	9.2	233.64	306.72	40.38242393	169.64	261.28	49.51377932	-0.36	198.72	90.10379676	N
16 (L)	M	68	Notched	2	50.5	50.9	11	15.2	10.8	235.4	334.4	45.25543384	226.68	328.32	46.33632033	6.6	237.6	88.40824582	N
16 (R)	M	68	Notched	2	49.7	50.1	10.3	15.7	10.5	242.33	323.42	41.47252411	250.65	329.7	40.51494141	-30.15	216.3	98.01253448	N
17 (L)	F	90	Unknown	?	46.8	45.5	12.6	15.1	6.1	349.56	380.52	23.27227813	106.46	184.22	54.69730628	-32.04	153.72	102.0304156	N
17 (R)	F	90	Notched	2	42.1	41.9	12.9	14.1	7.7	305.93	363.78	32.7567458	91.69	217.14	65.02251188	26.89	198.66	82.22074232	N
18 (L)	M	76	Fan-shaped	3	47.2	47.7	12.2	15.6	8.4	321.64	380.64	32.3282893	165.08	262.08	50.95843485	-23.96	204.96	96.71327584	N
18 (R)	M	76	Notched	2	48.5	48.1	13	16.4	9.4	349.6	426.4	34.92647338	188.32	308.32	52.35306493	-11.6	244.4	92.72046169	N
19 (L)	M	73	Narrow	1	51.4	51.6	12.3	17.2	11.8	307.89	423.12	43.30892568	283.79	405.92	45.64292234	-5.31	290.28	91.04815199	N
19 (R)	M	73	Fan-shaped	3	49.2	50.2	12.6	16.1	11.5	285.72	405.72	45.23266846	232.7	370.3	51.06713196	31.8	289.8	83.70019958	N
20 (L)	F	88	Unknown	?	46.3	47.2	9.3	15.3	10	220.58	284.58	39.18524952	247.6	306	35.98700107	-47.6	186	104.8277494	N
20 (R)	F	88	Unknown	?	45.7	47.7	9.8	15.3	10.2	226.09	299.88	41.06760885	242.09	312.12	39.13770851	-34.01	199.92	99.79468264	N
21 (L)	F	73	Notched	2	43.9	45.4	11.9	15.2	9.5	282.4	361.76	38.68188577	179.68	288.8	51.52591009	0.82	226.1	89.79220415	Y
21 (R)	F	73	Notched	2	45.6	45.2	12.1	14.9	9.8	272.38	360.58	40.94018383	171.64	292.04	54.00407523	20.44	237.16	85.05574094	Y
22 (L)	M	71	Unknown	?	50.1	50.3	12.4	16.3	11.8	280.21	404.24	46.11784943	251.17	384.68	49.23694813	27.31	292.64	84.64520244	Y
22 (R)	M	71	Unknown	?	48.6	49.7	13.5	16.7	11.4	331.18	450.9	42.73614922	226.6	380.76	53.47841804	33.32	307.8	83.78543274	Y
23 (L)	F	79	Unknown	?	45.8	45.4	13.1	15.3	10.7	291.21	400.86	43.40930267	176.97	327.42	57.28242357	52.01	280.34	79.30827375	Y
23 (R)	F	79	Unknown	?	46.7	46	13.3	16.4	9.6	353.69	436.24	35.82883753	184.23	314.88	54.191356	0.09	255.36	89.97980647	N
24 (L)	F	78	Unknown	?	46.7	47.1	13.1	16.3	9.7	343.21	427.06	36.51919256	188.17	316.22	53.48306193	0.01	254.14	89.9977455	Y
24 (R)	F	78	Unknown	?	47.2	46.8	13.8	17.2	9.6	394.12	474.72	33.87909739	197.56	330.24	53.25665074	-13.24	264.96	92.86425187	Y
25 (L)	M	90	Unknown	?	50.3	51.1	13.6	16.8	9.9	369.19	456.96	36.10618313	195.29	332.64	54.04914207	0.73	269.28	89.8446748	N
25 (R)	M	90	Fan-shaped	3	52.1	53.8	14.1	15.4	9.6	343.81	434.28	37.65738226	130.51	295.68	63.80743987	53.81	270.72	78.53517786	N
26 (R)	M	87	Unknown	?	54.1	55.1	14.1	18.7	11.6	413.94	527.34	38.28316252	285.44	433.84	48.85716686	-16.32	327.12	92.85967063	N
26 (L)	M	87	Unknown	?	54	55.5	14.1	19.6	11.9	441.36	552.72	37.01064691	326.96	466.48	45.50002645	-43.74	335.58	97.48932664	N
27 (R)	F	86	Unknown	?	48.4	47.7	13.6	16.6	11.4	330.56	451.52	42.93685412	220.56	378.48	54.35565834	39.36	310.08	82.70748754	N
27 (L)	F	86	Unknown	?	45	46.1	12.7	17.1	11.8	314.46	434.34	43.61468999	270.36	403.56	47.93775449	8.12	299.72	88.44755552	N
28 (R)	M	40	Notched	2	47.8	48	12.4	17.1	12.3	294.88	424.08	45.94564299	289.94	420.66	46.42921299	12.64	305.04	87.62514403	N
28 (L)	M	40	Unknown	?	48.9	49.1	11.9	16.5	10.9	295.05	392.7	41.29368277	249.45	359.7	46.09262433	-11.83	259.42	92.6136929	N
29 (R)	F	72	Notched	2	47.9	48	13.5	17.6	10.2	387.97	475.2	35.27048209	231.55	359.04	49.84075604	-23.47	275.4	94.88876187	N
29 (L)	F	72	Fan-shaped	3	50.4	49.6	14.3	18.6	11.4	420.49	531.96	37.77202539	271.43	424.08	50.20487475	-11.51	326.04	92.02309986	N
30 (R)	M	84	Narrow	1	44.9	45.1	12.1	16.7	12	281.3	404.14	45.88937741	276.48	400.8	46.38410957	11.52	290.4	87.72651302	N
30 (L)	M	84	Narrow	1	45.1	46.3	12.5	16.7	11.5	302.89	417.5	43.49078843	254.89	384.1	48.4247421	9.61	287.5	88.08446947	N
31 (R)	F	81	Narrow	1	47.2	47.2	14.4	16.3	10.5	362.8	469.44	39.39077862	168.58	342.3	60.49549671	51.92	302.4	80.11372466	Y
31 (L)	F	81	Notched	2	48.6	48.1	14.9	17.3	10.7	406.81	515.54	37.89898847	191.77	370.22	58.80252565	37.21	318.86	83.29848588	Y
32 (R)	M	92	Notched	2	46.3	47.8	12.2	16.3	10.6	302.17	397.72	40.55734253	229.21	345.56	48.44795065	-4.49	258.64	90.99470683	N
32 (L)	M	92	Fan-shaped	3	48.7	49.3	12.1	17.1	11.4	308.86	413.82	41.72368256	275.96	389.88	44.94319118	-16.04	275.88	93.33312625	N
33 (R)	M	72	Notched	2	53.2	52.8	13.8	18.3	11.2	399.89	505.08	37.65196289	269.89	409.92	48.82228505	-19.01	309.12	93.52575206	Y
33 (L)	M	72	Narrow	1	54.3	54.5	13.5	17.4	11.6	350.45	469.8	41.75874566	255.07	403.68	50.81237804	14.05	313.2	87.4288763	Y
34 (R)	F	79	Notched	2	52.1	52.8	15.3	18.8	11.5	455.28	575.28	37.68290794	251.6	432.4	54.41791956	12.9	351.9	87.8991725	Y
34 (L)	F	79	Narrow	1	53.3	53.7	15.1	19.2	11.8	457.41	579.84	37.92131121	279.87	453.12	51.85520317	-1.39	356.36	90.22348562	Y
35 (R)	M	84	Narrow	1	56.7	56.9	15	20.1	12.1	482.6	603	36.83821334	325.42	486.42	48.00927327	-32.6	363	95.15251339	Y
35 (L)	M	84	Fan-shaped	3	55.6	55.7	15	19.2	12.3	442.35	576	39.82816686	294.93	472.32	51.35975758	7.65	369	88.81207556	Y
36 (R)	M	83	Narrow	1	50.2	50.5	12.8	17.8	9.4	392.32	455.68	30.57601375	241.36	334.64	43.84199812	-64.64	240.64	105.5819881	Y
36 (L)	M	83	Fan-shaped	3	50.8	51.7	14.1	16.4	11.8	328.53	462.48	44.73531001	209.39	387.04	57.24802146	69.09	332.76	78.01666853	Y
37 (R)	F	84	Notched	2	46.8	47.1	11.7	16.2	11.7	262.44	379.08	46.18693854	262.44	379.08	46.18693854	11.34	273.78	87.62612292	N
37 (L)	F	84	Notched	2	46.6	47.5	11.7	15.9	10.8	273.06	372.06	42.7844797	232.56	343.44	47.37875636	0.72	252.72	89.83676394	N
38 (R)	M	86	Notched	2	49.9	51.4	12.5	18.3	11.3	363.45	457.5	37.39890487	306.33	413.58	42.21071618	-50.95	282.5	100.3903789	Y
38 (L)	M	86	Notched	2	50.6	52.1	12.3	18.2	10.6	370.17	447.72	34.22970361	292.31	385.84	40.74746879	-67.59	260.76	105.0228276	Y
39 (R)	F	99	Fan-shaped	3	49.7	48.3	13.7	17.4	10.8	373.81	476.76	38.36587589	231.71	375.84	51.93810761	1.57	295.92	89.6960165	N
39 (L)	F	99	Fan-shaped	3	48.6	49.7	15.4	17.3	9.7	442.36	532.84	33.88143848	156.22	335.62	62.25955699	31.96	298.76	83.85900453	Y
40 (R)	M	73	Narrow	1	47.3	48.2	11.9	17.2	11.3	309.76	409.36	40.82644607	281.92	388.72	43.51017559	-26.54	268.94	95.66337834	N
40 (L)	M	73	Narrow	1	47.5	48.6	12.1	16.9	10.4	323.86	408.98	37.639158	247.36	351.52	45.2764463	-31.04	251.68	97.08439571	N
41 (R)	F	81	Fan-shaped	3	49.3	47.4	14.9	15.9	8.9	395.61	473.82	33.39068812	110.01	283.02	67.12633855	48.41	265.22	79.48297332	Y
41 (L)	F	81	Unknown	?	47.9	46.5	14.8	15.3	9.8	357.09	452.88	37.95565522	111.09	299.88	68.25673969	80.99	290.08	73.78760509	Y
42 (R)	M	81	Notched	2	45.5	45.4	12.8	16	12	275.84	409.6	47.66706966	236.16	384	52.04807971	51.84	307.2	80.28485063	Y
42 (L)	M	81	Notched	2	46.3	47.1	13	15.8	11.4	288.68	410.8	45.3538462	210.6	360.24	54.22453857	49.32	296.4	80.42161522	Y
43 (R)	F	75	Fan-shaped	3	46.9	47.7	12.1	16.2	9.5	318.6	392.04	35.64216203	206.28	307.8	47.91939332	-25.78	229.9	96.43844465	Y
43 (L)	F	75	Fan-shaped	3	46.9	46.6	12.1	16.7	10.3	319.21	404.14	37.82849917	238.57	344.02	46.09401248	-26.39	249.26	96.07748834	Y
44 (R)	M	81	Notched	2	53.9	54.6	13.3	17.8	11.6	359.17	473.48	40.66128332	274.51	412.96	48.33780937	-5.39	308.56	91.00090731	Y
44 (L)	M	81	Notched	2	57.7	57.8	14	18.7	10.2	441.65	523.6	32.48976688	257.73	381.48	47.49880875	-49.65	285.6	100.0114244	Y
